# Planned Obsolescence in the Context of a Holistic Legal Sphere and the Circular Economy

**DOI:** 10.1093/ojls/gqaa061

**Published:** 2021-02-07

**Authors:** Jurgita Malinauskaite, Fatih Buğra Erdem

**Keywords:** planned obsolescence, circular economy, consumer protection and unfair competition, competition law, environmental law

## Abstract

Businesses may take advantage of rapid technological developments to increase sales by designing products with a short lifespan and encouraging consumers to buy a replacement more quickly than they otherwise might have to, which is commonly known as planned obsolescence. While employing a holistic approach and exploring planned obsolescence from three different angles—the demand side, supply side and environmental side—the article argues that the current measures in the fields of unfair competition and consumer protection law, competition law and environmental law are quite insufficient to deal with planned obsolescence. Therefore, there is a need for an EU measure outlawing planned obsolescence in the context of the circular economy.

## 1. Introduction

A circular economy (CE) is the EU’s main strategy for replacing linear ‘take–make–use–waste’ models with more circular ones, where resources are used and kept for longer to reduce and avoid waste as much as possible. In 2015, the European Commission adopted its first ambitious Circular Economy Action Plan.^1^ This included steps to stimulate Europe’s transition towards a CE, embracing measures covering the whole cycle, from production and consumption to waste management and the market for secondary raw materials, thus contributing to ‘closing the loop’ of product life cycles through greater recycling and reuse, and bringing benefits for both the environment and the economy.

While a product’s lifespan depends on a network of interdependent stakeholders (the manufacturers, suppliers, distributors and consumers), and can be dictated by uncontrolled factors, such as ‘the wear and tear of the materials’, quite often it is the result of entrepreneurial choices.^2^ Businesses may take advantage of the rapid technological and scientific developments in product manufacturing to increase their sales and profits by using various strategies, including the so-called ‘planned obsolescence’. The intention of applying planned obsolescence is to design products with a short lifetime and to encourage consumers to buy a replacement more quickly than they might otherwise have to. Therefore, product design plays a crucial role in terms of the product’s lifespan, which is reinforced by advertising, incompatibility^3^ and poor aftersales services.

Historically, planned obsolescence has been widely accepted and even promoted as part of the ‘Throw-Away Society’ campaign, championed most prominently in the United States but with an impact across Europe, which has enabled businesses to preserve a high volume of sales through advertising to instil in consumers the desire to own something ‘a little newer, a little better, a little sooner than is necessary’^4^ since the 1950s. While it is debatable the extent to which this strategy presented the most efficient solution to overcome the economic standstill after World War II,^5^ this culture is no longer sustainable with a global population of nearly eight billion.^6^

Planned obsolescence directly contradicts the concept of a CE due to its promotion of a culture of wastefulness by perpetuating a ‘buy new and buy often’ mentality, leading to harmful effects on the environment. Detrimental implications of planned obsolescence have not gone unnoticed, but have attracted considerable attention from several disciplines, including engineering,^7^ economics^8^ and environmental studies.^9^ Yet, legal studies have been somewhat limited until recently.^10^ The popularity of planned obsolescence has increased following investigations into the practices of Apple and Samsung in France and Italy mainly for violations of the provisions on unfair competition and consumer protection. In contrast to the previous literature,^11^ this article employs a holistic approach and positions planned obsolescence in three main areas, namely unfair competition and consumer protection law, environmental law^12^ and competition law,^13^ which have been employed or could potentially be employed to deal with planned obsolescence. These are underpinned by economic considerations from the demand side, the supply side and the environmental side concerning their impacts on the market, seeing planned obsolescence as a specific type of market failure. The article argues that the current EU measures in these fields are not sufficient to deal with the planned obsolescence issue,^14^ and that there is therefore an urgent need to have strict measures at the EU level banning planned obsolescence. The EU action is justified as it relates directly to the internal market, especially as it is supported by the CE concept. Building on this connotation, this article further argues that the EU-driven strategy towards a CE is superfluous where the durability of products is curtailed by unregulated planned obsolescence. To strengthen this argument, some insights from rule utilitarianism and its notion of responsibility for sustainability have been sought. While this article focuses predominantly on electronic and electrical devices, it is also applicable to other products, such as clothes, toys and automobiles.^15^

The article is structured as follows. Following this introduction (section 1), sections 2 and 3 give insight into the history of planned obsolescence and the multifaceted concept of planned obsolescence, respectively. Section 4 then presents planned obsolescence from a holistic perspective through demand-side, supply-side and environmental-side viewpoints, whereas section 5 reflects on the EU’s CE strategy by considering the ‘rule utilitarianism’ approach and intergenerational justice. Section 6 then analyses the extent to which the current EU provisions in the fields of unfair competition and consumer law, competition law and environmental law cover planned obsolescence. Divergent national approaches to deal with planned obsolescence employed by the selected EU Member States and beyond are discussed in section 7. The EU developments concerning planned obsolescence are subsequently provided in section 8, with concluding remarks and the way forward being presented in section 9.

## 2. Planned Obsolescence in a Historical Context

The issue of planned obsolescence has been examined under different disciplines according to the problems of specific epochs. Historically, traces of planned obsolescence in the United States can be found as early as in the 1870s in the context of disposable shirt collars,^16^ but it did not rise to prominence on the national level until the electric starter in the 1912 Cadillac Touring Edition, which made all previous cars obsolete.^17^ Planned obsolescence was also visible in industries other than the automobile industry, the most notable example from the early years being light bulbs,^18^ which led to the Phoebus cartel in 1925. In this case, the businesses agreed to intentionally limit the lifetime of light bulbs to 1000 hours, with corporate resolutions to modify the bulbs’ filaments.^19^ Another example is the strategy employed by DuPont^20^ to produce fast-wearing nylon stockings, which were easily torn or stretched, leading to a high number of sales throughout the 1950s and beyond.^21^

After consecutive wars in the 20th century, both global and national economies had come to a standstill, leading to the Great Depression. Planned obsolescence was seen as a way out of the 1929 economic crisis for the US Government, which could collect more tax with increased transactions in the United States. Indeed, the real estate agent Bernard London, in his eminent article where the concept of planned obsolescence was first defined, claimed that public and individual expenditures must be increased to create a more sustainable economic system by improving deficient demand through applying planned obsolescence strategies, with every commodity, including buildings and machinery, being declared obsolete after an allotted time by banning their subsequent use. This was regarded as a way to provide ‘a new reservoir from which to draw income for the operation of the government’.^22^ This strategy was therefore employed to augment the volume of sales to boost the economy, as previously those goods had been built to last a lifetime.^23^

Apart from adjusting the product design, it was also necessary to change consumer consumption culture. Adorno and Horkheimer, accordingly, argued that every consumer could be manipulated by the culture industry to buy certain goods even if they did not need them.^24^ Therefore, planned obsolescence was seen as a strategy of causing products to become obsolete before the end of their actual lifetime.^25^ This was reinforced in the 1950s on both sides of the Atlantic by the ‘Throw-Away Society’ campaign for consumers to own something ‘a little newer, a little better, a little sooner than is necessary’;^26^ since using fashionable products has become an important indicator of human relationships, indicating some sort of ‘status’ in society, this appertains directly to planned obsolescence by its very nature. Moreover, the throw-away culture was also adopted by producing disposable products such as batteries, cartridges, pens and razors.^27^ Nevertheless, the scholars seem to doubt the extent to which planned obsolescence was an efficient way to overcome the economic crisis.^28^

## 3. Concept of ‘Planned Obsolescence’

Starting with London’s breakthrough concept, planned obsolescence has traditionally been described as the intentional production of goods and services with lifespans limited by stimulating consumers to repeat their purchases too frequently.^29^ The lifespan here refers to the ability of a product to function at the anticipated performance level over a given period (ie the number of cycles; hours in use) under the expected conditions of use and pursuant to foreseeable actions’.^30^

Different terminology has been used in the literature to define the multifaceted notion of obsolescence, including premature, planned or purposeful obsolescence and built-in obsolescence, with further configurations of physical obsolescence, technological obsolescence, style obsolescence and psychological obsolescence. For consistency, this article categorises planned obsolescence into two main groups: (i) technological obsolescence (which also includes physical obsolescence); and (ii) style (or psychological) obsolescence. Under the first category, producers deliberately design products with a shorter physical life than the industry is capable of producing under existing technological and cost conditions.^31^ This would include a process whereby producers artificially limit the durability of a product in order to stimulate repetitive consumption, or design it in such a way that it functions for only a limited number of operations.^32^ This category also embraces designing for limited repair: it could be because the component required for repair is unobtainable or it is simply not practical or worth repairing the product. Furthermore, there is also incompatibility obsolescence (also known as technological obsolescence), where the invention of a technically advanced product makes the previous models obsolete,^33^ for example, by introducing non-compatible software updates or voluminous updates which necessitate consumers purchasing the newer version of the product. In this context, whether product lifespan reduction has occurred before products are placed on the market^34^ or afterwards is relevant. The latter is closely linked not only with incompatibility obsolescence, but also with the second category of style obsolescence, which defines scenarios where manufacturers or sellers induce consumers to replace goods even though they still retain substantial physical usefulness.^35^ This is related to marketing campaigns and is based on the consumer’s perception, rather than the product itself. This is mainly because of different ‘fashion’, different colours, changed styles or the addition of an extra function to a model, which makes the appearance of previous models ‘old’ and undesirable. As previously suggested, in this description, technological obsolescence can overlap with psychological obsolescence: producers that release technically updated products might persuade consumers who are vulnerable to ‘status’ to buy the technically newer or better product, whereas the ‘old’ product still functions properly. This psychological obsolescence strategy was used, for example, by General Motors as early as in the 1920s, when it boosted its sales solely on the improved design of their cars.^36^

While there is some understanding on what planned obsolescence entails, a legal definition is lacking. This creates legal uncertainty, as it is not clear whether all these different nuances of planned/style obsolescence should be treated and addressed equally, or the legislator requires different approaches. There are also uncertainties as to whether specific features of different product groups (ie electronic and electrical devices, toys, clothes, etc) should also be considered and whether ‘more tailored’ regulations should apply. This thus suggests the need for an EU-level definition, which is largely supported by the European Parliament.^37^

## 4. Planned Obsolescence from a Holistic Perspective

This article addresses planned obsolescence from a holistic approach, positioning it into three main areas, namely unfair competition and consumer protection, competition law, and environmental law, which traditionally are underpinned by economic considerations respectively from the demand side, the supply side and the environmental side concerning their impacts on the market.

### A. Demand Side

Adam Smith, in his eminent theory of the invisible hand, described the workings of a market economy, which involves consumers deciding to buy certain goods and thus rewarding the firms that produce those goods, whereas firms that cannot win over consumers either have to improve their goods or leave the market.^38^ Further, the consumer sovereignty theory was developed by the economist Ludwig von Mises, whereby the economy acts primarily in response to the aggregate signals of consumer demands.^39^ The essence of consumer sovereignty is the exercise of choice, which incorporates two elements: (i) the availability of options^40^ in the marketplace; and (ii) the ability of consumers to choose freely among these choices.^41^ Given that the consumer sovereignty is associated with a rational person (who chooses goods which maximise her utility), it is worth mentioning insights of behavioural economics, as behavioural economics notes that consumers usually make irrational decisions.^42^ This is because people possess ‘bounded rationality’.^43^ There are striking empirical findings on the decision-making mechanisms of consumers showing that consumers can be easily misdirected in certain circumstances.^44^ In the context of the retail energy sector,^45^ Siciliani, Riefa and Gamper argue that consumers face artificially high search costs, due to the cognitive overload triggered by the amount and complexity of information about pricing that needs to be considered to make fully informed purchasing decisions (this can lead to a market failure, a phenomenon known in the literature as ‘confusopoly’).^46^ Under confusopoly, rival businesses avert cut-throat price competition by marketing their offering in various confusing ways to make it more difficult for consumers to shop around effectively.^47^ Building on this line of thought, consumers could be manipulated to buy products even though there is no need for them. As discussed above, planned obsolescence induces ‘consumer disposal behaviour’, which can contribute to an individual’s financial poverty through an increase in credit purchases and consumer indebtedness, especially among the most vulnerable disadvantaged groups.^48^ Studies^49^ have shown that the obsolescence of a functional product is more likely to affect consumers with a lower income than those with a higher income. For example, businesses encourage consumers to upgrade their mobile phones at sequential time intervals by purchasing a newer model. Given the general trend of price increases of new models due to inflation and the implementation of newer technology, not everyone can afford this. Planned obsolescence, therefore, puts greater financial pressure on those with lower income, potentially lowering the quality of life by reducing their financial freedom, as opposed to those with higher incomes, who are able to upgrade without feeling any financial pressure.^50^ Furthermore, due to the planned obsolescence practice, consumers’ trust in the market can be eroded as a result of dissatisfaction with the low quality and limited lifespans of products.^51^

### B. Supply Side

Under producer sovereignty, producers employ persuasive advertising techniques to entice consumers to buy what they wish to sell. Therefore, producers are not merely responding to consumer demand, but are deliberately influencing market conditions.^52^ When producers bring products to the market, under a simplified model, every commercial product goes through four stages: introduction, growth, maturity and decline.^53^ In the introduction phase, the product does not yield a profit, but in the growth phase, the profit will increase more with the lack of competition.^54^ During the maturity period, significant parameters, such as brand loyalty and consumption habit, become an issue.^55^ Therefore, the producer should find a way to replace or modify the product before the end of the maturity phase.^56^ Maintaining a high rate of growth is a challenge, and durable products can even exacerbate this problem.^57^ To avoid a decrease in sales, producers can manipulate a product lifespan via planned obsolescence,^58^ thus enabling businesses to increase their revenues through faster replacements. One can argue that planned obsolescence may also increase innovation, as durable products may make markets become too saturated. The rapid pace of innovation would likely shorten the product lifespan, as manufacturers require the continuous implementation of successful innovations to compete in innovation-driven markets. Economists warn that a broader discussion on optimal quality of products is essential based on the holistic life-cycle assessment of alternative product quality options.^59^ Businesses should choose the optimal longevity and quality for their products based on consumers’ expectations (the quality for which consumers are willing to pay) and the rate of innovation. Furthermore, producers with a large market share can have a competitive advantage from costly product update changes against their competitors, especially small competitors (eg small- and medium-sized enterprises (SMEs)),^60^ as adopting new market trends has a cost that SMEs may not be able to afford. This, in turn, may not only cause a loss for current competitors, but also create market entry barriers for new businesses. Therefore, not all businesses can benefit from planned obsolescence practices.

### C. Environmental Side

The problem of planned obsolescence not only inconveniences and burdens consumers and competitors financially, but is also detrimental to the environment, as it increases the amount of industrial waste (especially if the products are impossible or too expensive to repair, so consumers are forced to purchase replacements). There is an increasing trend of electrical and electronic equipment (EEE) production and sales in the EU, but with the lifetime use of the equipment decreasing.^61^ Subsequently, the quantity of waste of electrical and electronic equipment (WEEE) generated has recently expanded, which has contributed to the follow-on cost of planned obsolescence.^62^ Indeed, WEEE, such as computers, televisions, fridges and cell phones, is one of the fastest growing waste streams in the EU, with approximately 10.1 million tonnes in 2016 (an increase of 2.9% from 2015),^63^ and is expected to grow to more than 12 million tonnes by 2020.^64^ It has been noted that approximately 80% of the pollution and 90% of the manufacturing costs associated with EEE are the result of decisions made at the product design stage.^65^ The WEEE waste stream contains a high incidence of permanent toxins, such as arsenic, cadmium and radioactive substances,^66^ which are deleterious to the health of people living near to such waste and may cause airborne diseases. The treatment of WEEE is also complex and varies enormously according to the category of WEEE and the technology used, as EEE products are manufactured from different components and materials.^67^

The impact of WEEE goes beyond the EU, as most illegal electronic waste from the EU ends up in underdeveloped countries.^68^ While it is illegal to export e-waste containing toxic substances from EU to non-OECD and non-EU countries, EU countries nevertheless ship approximately 352,474 metric tonnes of electronic waste every year to developing countries.^69^

Furthermore, planned obsolescence is responsible not only for the increase in WEEE, but also for increased usage of natural resources (including critical raw materials). Smartphones, for example, contain a number of rare metals.^70^ Chile is the second-largest producer of lithium (after Australia), which is used for producing batteries for smartphones, laptops and electric cars. Given that demand for lithium has soared in recent years due to numerous perpetual productions, with global output rising threefold since 2005 to 85,000 tonnes in 2018,^71^ there is a further environmental impact in Chile in the form of drought (due to fresh water being used in the process), and consequently changes to the ecosystem.^72^ Therefore, the intergenerational argument is indispensable in this context, and will be explored in the following section.

## 5. The EU Circular Economy Strategy with Support from the Utilitarian Theory and Intergenerational Justice

In 2015, the European Commission adopted a Circular Economy Action Plan, with the aim to ensure that ‘nothing is wasted’. It included measures to encourage Europe’s transition towards a CE, to boost global competitiveness, to foster sustainable economic growth and to generate new jobs.^73^ The idea is to ‘close the loop’ of product life cycles, which consist of the extraction of materials (resources), the design and manufacture of products, their distribution and usage to what is considered waste, with further recycling and reuse^74^ to bring the product back to the market, thereby benefiting the environment and the economy. In 2020, a new Circular Economy Action Plan was issued which calls for further circularity to decouple economic growth from resource use.^75^ Planned obsolescence directly contradicts this strategy due to its deliberate shortening of a product’s physical life or convincing consumers that their products are obsolete before the end of their physical lifetime. Therefore, the CE’s objectives cannot be achieved without the regulation of planned obsolescence.

Since sustainability is one of the main goals of the CE, insights into a utilitarian notion of responsibility for sustainability are beneficial. Utilitarianism constitutes the philosophical basis of this study because it theoretically plays a binding role between law, economy and the environment.^76^ While without criticism, the utilitarianism theory, systematised by Bentham, is based upon pain aversion and pleasure maximisation. Generally speaking, according to the theory, the law should provide an overall effect for the greatest happiness of the greatest number,^77^ as utilitarianism is preoccupied not only with people’s welfare (*utilitas* in Latin), but, more importantly, by a fair organisation of society, which maximises the aggregate welfare of its members.^78^ Furthermore, the ‘rule utilitarianism’ approach argues that acts should provide the greatest utility to the greatest number of people, and those utilities should be beneficial to the society in the long run.^79^ Building on this approach, this article conceptualises utilitarianism in two different scenarios. First, if people wish to constantly purchase new products even if their old products function perfectly, they need to have more income and therefore they need to work more and harder.^80^ Life becomes dominated by work and in this spiral (rat race) life satisfaction (or happiness, as measured by utilitarians) is no longer increasing, even though income increases. Therefore, planned obsolescence and the fast replacement of products would not be justifiable under this utilitarianism approach. Secondly and most importantly, since humans’ existence is contingent upon a sustainable environment, considering environmental protection would be regarded as both intrinsically and instrumentally right.^81^ In the context of sustainability, a requirement for intergenerational justice, which is strongly supported in this article constitutes ‘one of its key components’.^82^ It is generally accepted that societies are ultimately responsible for the welfare of the next generations by assuring ecologic balance^83^ and basic sustenance for the progress of humankind.^84^ Pursuant to utilitarians, savings (in generational terms) are not only authorised, but are required, since the goal is to maximise the size of the intergenerational ‘pie’. Furthermore, Singer’s principle states that ‘if it is in our power to prevent something bad from happening, without thereby sacrificing anything of comparable moral importance, we ought, morally, to do it’.^85^ Based on Singer’s principle, Funfgelt and Baumgartner conclude that the utilitarian notion of responsibility for sustainability implies that it is ‘the responsibility [of everyone] to use [their] power to meet the needs of the present generation without compromising the ability of future generations to meet their needs’, provided that the current generation is not deprived of their basic needs.^86^ Every production has a cost (pain) for the environment because it entails the consumption of environmental assets. That is why disposable, non-recyclable, irreparable and easily perishable products as a result of planned obsolescence strategies will jeopardise the environment,^87^ which, in turn, will inevitably deprive the future generations of the ability to fulfil their needs.

## 6. EU Legislation Related to Planned Obsolescence

Currently, there is no EU legislation directly forbidding planned obsolescence. Rather, different types of legislation are employed that indirectly deal with planned obsolescence, namely consumer protection and unfair competition, competition law and environmental law. These are further explored in the following subsections.

### A. Unfair Competition and Consumer Protection 

Planned obsolescence can, under certain conditions, be considered as a breach of the Unfair Commercial Practices Directive (UCPD),^88^ the Consumers Rights Directive (CRD)^89^ or the Consumer Sales and Guarantees Directive (CSGD), since a lack of conformity with the contract (eg a defect) gives consumers legal guarantee rights.^90^ The leading consumer concerns are design features that do not allow repair by using digital locks or copyrighted software, upgradability or interoperability with other devices; the unavailability of spare parts and high repair costs; and marketing strategies pushing consumers to buy new, fashionable products and replace existing ones very quickly.^91^ Additionally, quite often clauses in user agreements are included where consumers (often unintentionally) agree not to fix their own products. While some consumers perceive planned obsolescence as a natural course of action that goods have a short lifespan, others are infuriated by it due to a lack of business ethics, environmental concerns and disregard for consumer rights. Pursuant to a 2014 Eurobarometer survey, 77% of European citizens would prefer to repair their goods rather than buy new ones, but ultimately have to replace or discard them because they are discouraged by the cost of repairs and the level of service provided.^92^

As per the CRD, consumers expect that the goods delivered to them conform to the contract. This directive also imposes on the seller or trader the obligation of providing in a clear and comprehensible manner, and prior to the conclusion of the contract, sufficient information regarding the main characteristics of the goods/services, the existence of guarantees of conformity for goods, aftersales services, the functionality of any digital content, and the interoperability of any digital content with other hardware and software. Therefore, prior to the conclusion of the purchase agreement, consumers must be clearly and sufficiently informed about the functionalities and life cycle of products.

In case of non-compliance with the contract, such as a defect that becomes apparent within two years from the delivery of the product, the consumer can invoke the legal guarantee under the CSGD.^93^ For second-hand products, a shorter liability period can be agreed, but not less than one year.^94^ Therefore, consumers can ask for the products to be repaired, replaced or reduced in price, or for the contract to be terminated. Given that these clauses provide for minimum harmonisation, Member States could set their own, longer warranty periods. The majority of the Member States followed the directive verbatim.^95^ In addition, most EU countries require consumers to inform the seller of the lack of conformity within two months after its discovery.

The fact that the right to repair is enforceable up to the expiry of the warranty is of relevance in cases of planned obsolescence, as one can claim that businesses manipulate their products’ lifespan to coincide with the end of the warranty period, by designing their goods in such a way that they malfunction shortly after the warranty period lapses.^96^

The Commission has also recently revised the CRD; the changes thereto include, *inter alia*, a measure aimed to prioritise further the right to repair. For instance, recital 48 states that while consumers should enjoy a choice between repair or replacement, ‘Enabling consumers to require repair should encourage sustainable consumption and could contribute to greater durability of products’.^97^ Yet, some scholars have noted that the directive’s text prefers the strict hierarchy of remedies that might facilitate easier termination of contracts.^98^

Planned obsolescence can also be regarded as an unfair commercial practice, covered under the UCPD, which aims to boost consumer confidence and create a level playing field of competition between producers within the internal market in the EU, to make it easier for businesses, especially SMEs, to trade across borders by enabling national enforcers to curb a broad range of unfair business practices, such as untruthful information to consumers or the use of aggressive marketing techniques to influence their choices. The UCDP is aimed at maximum harmonisation, which technically means that Member States cannot regulate or prohibit the practices defined by the directive.^99^ Yet, the European Commission noted in its guidelines that the UCDP does not relate to national rules that are not of a commercial nature. Therefore, more stringent rules can be adopted at the national level to regulate commercial practices with regard to the protection of health, safety and the environment.^100^ The UCPD blacklists practices that are to be regarded as unfair practices under any circumstances. Yet, it does not include a technique to shorten a product’s lifespan.^101^ Apart from blacklisting practices regarded as ‘unfair’, the UCDP also contains a general clause of unfair commercial practice and more specific provisions on misleading (or aggressive) practices which are classified as misleading actions (article 6 UCDP) or misleading omissions (article 7 UCDP). Previous studies have dismissed the application of article 6 UCPD due to its unsuitability to cover planned obsolescence;^102^ thus, the emphasis is placed on article 7, which proclaims that

A commercial practice shall be regarded as misleading if … taking account of all its features and circumstances and the limitations of the communication medium, it omits material information that the average consumer needs, according to the context, to take an informed transactional decision and thereby causes or is likely to cause the average consumer to take a transactional decision that he would not have taken otherwise.^103^

In this context, the commercial practice is misleading if it omits the ‘material information’, which can be summarised as follows: the main characteristics of the product, the identity of the trader, the price, freight, delivery charges, arrangements for payment, delivery, performance, existence of a right of cancellation etc. This notion is strictly interpreted by the Court of Justice of the European Union, as it also includes how information is provided.^104^ Therefore, if a trader fails to inform consumers that a product has been designed with a limited lifetime, it might be considered to have omitted to provide material information. In its recent guidelines, the Commission further notes that if a telephone’s battery (which is subject to particular wear and tear) cannot be replaced, or the functional lifetime of a refrigerator is planned to be significantly shorter than comparable products, it could be a breach of article 7 UCPD.^105^

Finally, due to the information asymmetry, consumers find it difficult to prove that producers acted deliberately to create a necessity for replacement or renewal, which is further reinforced by the ability to invoke business secrecy or to protect their patents.^106^ Therefore, consumer protection measures are not without shortcomings. The article also covers only business-to-consumer relations; therefore, business-to-business relationships are left out, which will be discussed next in the context of competition law.

### B. Competition Law

On the supply side, competition law can also play a role in dealing with planned obsolescence.^107^ The traditional EU theory of harm is connoted to consumer welfare linked to three aspects: quality, price and choice (also known as innovation). Planned obsolescence is a complex multivariable process, which causes some tensions in terms of competition law. On the one hand, planned obsolescence can spur an increase in R&D investments and the rate of innovation, leading to an increase in consumer welfare.^108^ One may argue that it is hard to make room for innovative products so long as consumers continue to use their older durable products. On the other hand, it may worsen consumer debt levels and reduce consumer welfare due to poor quality and short-lived products.^109^ It should be noted that price is correlated to quality, though this relationship is complex and consumers do not always use price as a surrogate measure of quality.^110^ For instance, price-sensitive consumers buy the cheapest products regardless of their quality and lifetime. A short lifespan of a product could also be a matter of price and quality (a product made from low-quality materials). Against this background, pricing low-quality products with a short lifespan at a lower level on the market would increase consumer welfare by meeting the needs of the price-sensitive consumers. However, it is doubtful whether these vulnerable consumers benefit in the long run, as cheaper products that break easily leave them with no choice but to repurchase the same products.

The concept of planned obsolescence accommodates different scenarios when analysed in monopolistic, oligopolistic and competitive markets.^111^ The monopolist would likely desire to reduce durability in order to reap monopoly profits, engage in tying practices and obstruct its competitors from entering the market or harming its sales. An oligopoly can create an environment that gives the oligopolists an incentive to collude in reducing the durability of their products to an inefficient level; as a side effect, it will also consider the threat to the stability of the cartel posed by the increased frequency of interactions in the market. In terms of finding suitable market conditions for planned obsolescence, it has been revealed that planned obsolescence is only effective in monopolistic and oligopolistic markets. Under competitive market conditions, employing planned obsolescence would harm businesses.^112^ Moreover, it has also been proved that manufacturers of durable goods are comparatively weaker than manufacturers of perishable goods.^113^ From the microeconomics perspective, producers have incentives to limit their products’ lifetime to prevent plenty of used products from flooding the market.^114^ On the other hand, from the macroeconomic perspective, consumerism and capitalism are interwoven concepts that hasten the consumption cycle by encouraging manufacturers to adopt built-in obsolescence strategies and use fashion, advertising and other tools for increasing the number of sales.^115^

As far as specific provisions are concerned, article 101 TFEU (or domestic equivalents in the EU Member States) prohibits agreements and the association of agreements and concerted practices that have as their object or effect restriction of competition. Therefore, article 101 TFEU could impose the fining of undertakings if they decide to worsen the product quality by lessening the products’ lifespan (as was the case with the Phoebus cartel mentioned above) while not reducing the price.^116^ However, if it is the intention of a cartel to not only worsen the quality but also to significantly reduce the price in order for innovative products to be available to the wider public (expanding availability to those consumers who previously could not afford it), proving an infringement of article 101 TFEU will be close to impossible.

The application of article 102 TFEU in the context of planned obsolescence is not straightforward either. First of all, article 102 TFEU covers solely dominant players that abuse their positions on the market. Some business strategies employed by dominant players can be regarded as ‘abusive’ and fall under article 102 TFEU, as dominant undertakings have a ‘special responsibility’ towards the competitive process.^117^ Article 102 TFEU lists four explicit examples of potential abuse, which are purposely designed for broad applications.^118^ Clearly, this list is not exhaustive and new types can be identified, as the *AstraZeneca* case illustrates.^119^ As with consumer protection and unfair competition, the problem of planned obsolescence in competition law also stems from information asymmetry, as producers have information about product quality (ie product lifespan) not available to the consumer. Planned obsolescence can incite anticompetitive impacts in both primary and secondary markets. Aftermarkets become more of an issue for both complex technological products manufacturers and independent service organisations because aftermarkets accommodate a significant market share for either repairing or upgrading hardware or software of products.^120^ There are big conflicts between manufacturers, downstream market players and consumers in this arena. One of the abusive business practices could be related to a lock-in issue with halting the production of essential parts for the operability of the old products and designing programs incompatible with the old software systems. Likewise, the high costs of repairing or upgrading^121^ and the low value of the products’ second-hand market prices are also different aspects of the same strategy, which aims to sell new products to the consumer. For instance, in the *Pelikan v Kyocera*^122^ and *Info-Lab v Ricoh* cases,^123^ the Commission noted that consumers should be able to make an informed choice, including lifetime pricing of products, in advance. Hence, they need to be able to calculate the average cost of products. This information should cover aftermarket conditions. Due to high switching costs, consumers would likely be in lock-in situations most of the time.^124^ In theory, it is widely accepted that competition in the primary market prevents monopolisation in the aftermarket. However, this is not practically observable because of market failures, such as information asymmetry and brand switching costs. The influence of planned obsolescence practices in the secondary market would likely be within the EU competition law’s field of interest. For instance, in the *Qualcomm* case,^125^ the Commission found Qualcomm had abused its dominant position by becoming Apple’s sole supplier of long-term evolution baseband chipsets, which prevented Apple from dealing with Qualcomm’s competitors regardless of the quality of their chipsets due to the high switching costs associated with exclusivity. Therefore, by locking in Apple, Qualcomm foreclosed rivals from the market and stifled innovation. Switching cost also played a major role in the *Tetra Pak*,^126^ *IMS/NDC*^127^ and *Microsoft*^128^ cases. Thus, creating a switching cost is a strategic behaviour of dominant companies who wish to abuse their advantages.^129^

Planned obsolescence does not have to fit into any specific category defined by article 102(a)–(d) TFEU. Yet, building on *AstraZeneca*,^130^ the existence of any anticompetitive practices that would likely affect competition in the market is sufficient to warrant a competition law inspection. This makes inroads into intervening such cases even if other branches of law prohibit the same situations:

The fact that other laws and remedies prohibit misleading representations or provide for remedies against them is irrelevant where the objective of competition enforcement is not to penalise such misconduct per se, but rather to prevent the anticompetitive effects of such misconduct in the marketplace. Such anticompetitive effects must fall within the scope of competition law, and the fact that otherwise prohibited means may have been used to achieve them cannot be decisive for the application of competition law.^131^

The *AstraZeneca* case implies that competition law can apply to avert anticompetitive effects in the markets independent of enforcement of other laws.^132^ The legal precedent from this case illustrates that the application of article 102 TFEU has broad and extendable characteristics, which would likely enable planned obsolescence to be considered in this context. However, competition law does not provide a straitjacket solution either. While planned obsolescence could be regarded as anticompetitive practice and therefore would fall under article 102 TFEU prohibition, it is only applicable once dominance has been found. Given that competition law cases are complex and require in-depth economic analysis, proving planned obsolescence as anticompetitive practice (especially in the context of a trade-off between innovation and quality) could be challenging.

### C. Environmental Law

Planned obsolescence triggers and requires consumers to buy newer models of apparatus on a regular basis. Hence, it has not only economic but also environmental impact, as increased consumption inevitably leads to more waste. The concept of CE stresses the strong need to move up the waste management hierarchy. Article 4 of the Waste Framework Directive spells out the waste hierarchy, starting with the most preferred option of prevention, followed by reuse, recycling, recovery and, finally, the least preferred option—disposal.^133^ Building on this, designing more durable products would promote waste prevention, which should follow the stipulation of reuse, repair or refurbishment of products as a second option. For electrical and electronic equipment, the WEEE Directive also endorses the efficient use of resources and the retrieval of valuable secondary raw materials to ensure that precious resources are not lost.^134^ There are binding minimum collection targets imposed on the EU Member States for recycling, yet, unfortunately, there are no targets for the prevention of waste. Furthermore, the ‘polluter pays principle’^135^ and the ‘extended producer responsibility’^136^ were introduced to assign producers to long-term environmental responsibility. The idea is to internalise the waste externalities of products and provide incentives to convert from the linear ‘cradle-to-grave’ approach to the ‘cradle-to-cradle’ system, with further focus on how products are designed, marketed, used and reused embracing full life-cycle impacts. This has been mainly implemented through take-back schemes (ie requiring manufacturers to take back and recycle their products), deposit/refund schemes, labelling schemes, etc.^137^

To address planned obsolescence, the European Commission announced in its Circular Economy Action Plan a set of actions to be implemented, including requirements to support the durability and reparability of products.^138^ The EU Ecodesign Directive^139^ is a framework directive and defines the general principles of ecodesign, without placing any specific, restrictive conditions on manufacturers. The specific requirements are adopted through the regulations issued by the Commission for specific product groups. Various regulations have initially focused on setting binding energy efficiency requirements for the use phase of products, but the scope has since expanded to cover a wider range of environmental aspects. For instance, energy labelling requires a product to be labelled with a clear indication of the energy efficiency of products using a comparative scale from A (most efficient) to G (least efficient)—the concept that has been a key driver for helping consumers choose products which are more energy efficient, while simultaneously encouraging manufacturers to drive innovation by using more energy-efficient technologies.^140^ There is also the EU Ecolabel, which promotes the CE by encouraging producers to generate less waste and CO_2_ during the manufacturing process by developing products that are durable and easy to repair and recycle. Furthermore, new ecodesign rules were introduced that include measures to further enhance repairability and recyclability, improving the lifespan, maintenance, reuse, upgrading, recyclability and waste handling of appliances. It also embraces requirements such as ensuring the availability of spare parts—making essential parts more easily replaceable—and access to repair and maintenance information for professional repairers. Specifically, the ecodesign-implementing regulations were recently set for 10 product groups: refrigerators; washing machines; dishwashers; electronic displays (including televisions); light sources and separate control gears; external power supplies; electric motors; refrigerators with a direct sales function (for instance, fridges in supermarkets, vending machines for cold drinks); power transformers; and welding equipment.^141^ There is a plan to further expand the scope of these regulations.

The European Commission has also launched a study to analyse and develop a potential scoring system to rate the ‘ability to repair’ for products.^142^ For instance, France, in its roadmap on the CE, considers implementing a life-cycle index for products, which would consist of a note from 1 to 10 subject to the criteria of reparability, reliability or sustainability. In parallel, technical standards on material efficiency (including durability and reparability) are being developed by EU standardisation bodies.^143^ These standards can be used as a basis when setting product-specific standards.^144^ Furthermore, a call under the Horizon 2020 work programme has been published to prepare an independent testing programme on premature obsolescence.^145^ Based on the results of this research and other fact analyses, the Commission may consider follow-up initiatives, including horizontal legislation. Overarching regulation with horizontal application in this context would be significant since many technical regulations adhere to specific product groups, which may be too complex to comprehend for consumers to make informed choices.

While there are some encouraging measures in place, the ecodesign and energy-labelling measures do not expressly ban planned obsolescence and may be considered too ‘soft’ to prevent businesses’ practices, such as shortening their products’ lifespan or inducing consumers to buy new products without providing any alternatives. In addition, they are applicable solely to the specific categories of products.

## 7. National Developments in Dealing with Planned Obsolescence: Different Approaches across the EU Member States and Beyond

Given that currently there are no EU measures directly outlawing planned obsolescence, the Member States use various national laws (ie unfair competition and consumer protection, commercial law,^146^ contract law, environmental law). There were some unsuccessful attempts carried out by Belgium (in 2012 and 2018) and Italy (in 2013 and 2017) to introduce legislation against planned obsolescence.^147^ It seems that only France has succeeded in integrating the ban of planned obsolescence into its legislation. For consistency, this section will focus further on three main areas: environmental law; unfair competition and consumer protection; and competition law.

### A. Environmental Law

France banned planned obsolescence by the Energy Transition for Green Growth Act in 2015^148^ and set its sights on both economic growth and the decrease of environmental pollution.^149^ This Act initiated changes to the Consumer Code, where planned obsolescence, defined as ‘the use of techniques by which the person/entity responsible for placing a product on the market deliberately intends to shorten the life cycle in order to increase its replacement rate’,^150^ was outlawed and became a criminal offence punishable by a two-year prison sentence and a fine of up to €300 000 or of up to 5% of the company’s average turnover.^151^ The French Environmental association Hop! (‘Halte à l’obsolescence programmée’/’Stop planned obsolescence’) is proactively involved in filing claims in France against planned obsolescence. For instance, in 2017, it raised concerns that printer producers, namely Epson, Hewlett Packard (HP), Brother and Canon, have been taking advantage of specific techniques to decrease the lifetime of the cartridge by inserting sensors into their toner cartridges to stop them from working before they are actually empty (while more than 20% of the ink is still left).^152^ Most recently, in 2018, it also filed a complaint to the DGCCRF^153^ against Apple for intentionally decelerating its older smartphone models through software updates which led to a permanent slowdown of the system. Consumers were not well informed regarding the iOS operating system updates, namely 10.2.1 and 11.2, on their phone’s performance, and therefore were forced to purchase a new battery or new models. The DGCCRF subsequently fined Apple €25 million for its planned obsolescence practices by decreasing older iPhones’ performance (ie iPhone 6, SE and 7 models) and battery management.^154^

The other EU Member States are also looking for the best techniques to fight planned obsolescence practices. For instance, the Spanish NGO Amigos de la Tierra issued an initiative, *Alargascencia*, against obsolescence, by advocating the greatest possible prolongation of the useful life of products through the sale, rent and exchange of second-hand goods. Spanish Greenpeace has also launched a campaign to promote the better repair of mobile devices, as an antidote to the current tendency to buy new products all the time.^155^ Similarly, the Green Party in Germany shed light on planned obsolescence in 2013 through the institution of REGIO Stadt-und Regionalenticklung GmbH by preparing a report to eliminate all planned obsolescence-related practices to achieve zero waste by suggesting measures like standardisation and labelling about the life expectancy of items.^156^ Apart from this campaign headed by Schridde, the German Federal Environment Agency, the Swiss Federal Environment Agency and the Austrian Chamber of Labour have also raised the alarm about shrinking product lifespans.

### B. Unfair Competition and Consumer Protection 

The Italian Competition Authority (which is also the Consumer Protection Authority, Autorità Garante Della Concorrenza E Del Mercato, AGCM) also investigated planned obsolesce practices in the Apple and Samsung cases, namely for setting up ‘a general commercial policy taking advantage of the lack of certain components to curb the performance times of their products and induce consumers to buy new versions’. The AGCM deliberated that both companies consciously decelerated their smartphones by applying software updates without informing consumers: in particular, holders of Apple’s iPhone 6, 6 plus and 6s plus were induced to install an operating system specifically designed for the iPhone 7, leading to problems for owners of the older models; likewise, holders of Samsung’s Galaxy Note 4 were led to install a new version of Google’s Android operating system intended for the more recent Galaxy Note 7. As a result, the smartphones had become almost unusable and consumers were prevented from reverting to their previous statuses. This also had destructive effects on battery performance. While Apple acknowledged that it had intentionally slowed iPhones with degraded batteries through software updates to avoid sudden shutdown problems, it nevertheless denied other claims, including intentionally shortening the life of a product.^157^ The AGCM noted that both Apple and Samsung carried out ‘dishonest commercial practices’ and that operating system updates ‘caused serious malfunctions and significantly reduced performance, thus accelerating phones’ substitution’. Yet, it did not punish the release of the updates in both cases. Instead, the AGCM considered as unfair, misleading and aggressive those practices aimed at persistently signifying the requirement to update the smartphones’ operating system on the basis of information that concealed (or mystified) the negative impacts that the update would have on the performance of the smartphone, in particular on battery longevity, while precluding any means of restoring the previous functionality of the units.^158^ Because these practices were found unfair and misleading,^159^ Apple and Samsung were each fined the maximum amount allowed by law, which was €5 million. Furthermore, Apple was imposed an additional fine amounting to €5 million for failing to give consumers clear information about the characteristics of their batteries, including their average life expectancy, and how to maintain them (or eventually to replace them).^160^

### C. Competition Law

In terms of competition law, Apple has been and still is under various anticompetitive investigations by the European Commission^161^ and some Member States, such as France.^162^ Yet, there have been no cases in the EU directly dealing with planned obsolescence. Interestingly, in Europe, only the Russian Federal Antimonopoly Service (FAS, the Russian national competition authority) has investigated a potential violation—of the federal law ‘On Protection of Competition’^163^ by Apple Rus Ltd for its failure to ensure the use of goods within their life cycle, namely, the absence of supplies of components (ie screen modules and bother boards) required for repair in Russia, as a result of which the interests of consumers at large were infringed.^164^ The case was dismissed once Apple agreed to comply with the obligations imposed by the FAS to provide services for repairing/replacing screen modules on Apple smartphone models sold in Russia, which was half the price of replacing the old phone with a new one. In this case, there is a clear connotation to price and service in the aftermarket without any further considerations (ie environmental implications).

While there are widespread concerns about planned obsolescence across Europe and beyond, it seems that there is currently no consistency in tackling the issue, with countries taking different approaches. Only France has a clear, lucid ban on planned obsolescence, which has proved to be a success considering the 2020 Apple decision.^165^ The Italian decision against Apple and Samsung also provided a welcome outcome, even though the judgment inclined that if Apple and Samsung had been transparent and that consumers had been given a choice whether or not to replace their smartphones, then the obsolescence of those smartphones indirectly provided by the updates of their operating systems would not have been illegal. Different practices and regulations imposed on businesses by the Member States can also have detrimental effects on the internal market by distorting competition in the internal market. Therefore, a ‘one-note approach’ is necessary to address this issue. Articles 114 and 115 TFEU, accordingly, provide a legal basis for the EU to adopt measures approximating national rules which have the object of the establishment and functioning of the internal market. In this context, recourse to article 114 TFEU^166^ would be justified as banning planned obsolescence is instrumental to the achievement of the internal market, especially in the context of the CE.

## 8. Recent Developments on Planned Obsolescence in the EU

Planned obsolescence has not been totally ignored by the EU institutions. Notably, the European Economic and Social Committee (EESC)^167^ and the European Consumer Association have pushed the issue of planned obsolescence up their agendas and actively support legally binding measures to ban built-in obsolescence. The EESC was the first EU institution to raise the necessity of forbidding planned obsolescence, as it would be beneficial to the environment, consumers and, in the widest sense, the European economy.^168^ It stressed the multidimensionality of the problem that ‘designing products that become obsolete or break down prematurely is a major social, economic and environmental problem’.^169^ The EESC suggested new economic models under the CE system,^170^ which supports leasing or subscribing rather than possessing. It, accordingly, undertook a study^171^ aimed at discovering the most efficient way to use a product. For instance, consumers could have a chance to use a product when required without the necessity of owning the product. This, in turn, would place incentives on manufacturers to produce durable goods, given that they utilise products by employing rental business.^172^ The EESC further pinpointed implications on job opportunities. For instance, in the reuse and repair sector, the potential for job creation is estimated at 296 jobs for the equivalent of 10,000 tonnes of used goods. Given that a third of goods collected in waste recycling centres could be reused, this equates to over 200,000 local jobs that could be created if just 1% of municipal waste in Europe was prepared for reuse.^173^

Furthermore, the European Parliament, in a resolution about products having a longer lifetime that was adopted in July 2017, had also urged the European Commission to act to counter planned obsolescence.^174^ Specifically, it expresses the need for an EU-level definition of planned obsolescence for tangible goods and software; to examine the possibility of establishing an independent system that could test and detect the built-in obsolescence in products (in cooperation with market surveillance authorities); to provide better legal protection for whistleblowers; and to apply appropriate dissuasive measures against producers.^175^ To decrease environmental damage and to economically benefit consumers, the European Parliament strongly recommends designing upgradeable products.^176^ Specifically, it proposes several developments in the context of planned obsolescence. First of all, given the complex nature of planned obsolescence, the Parliament quite rightly calls for an EU-level definition regarding planned obsolescence on tangible goods and software. Secondly, there is also a need to create an independent mechanism to test and detect the planned obsolescence embedded in products. Finally, a mechanism for the reporting of planned obsolescence practices should be created simultaneously to provide better legal protection for whistleblowers.^177^

In the same resolution, the Parliament paid particular attention to the case of planned obsolescence caused by software updates, requiring, *inter alia*,

greater transparency as regards upgrading, security updates and durability; … definition of [a] reasonable period of use; clear information on the compatibility of updates and upgrades with embedded operating systems provided to consumers; … that the indispensable software updates are reversible and accompanied by information on the consequences on the operation of the device and that the new essential software is compatible with the previous generation software; … and … the modularity of the components, including the processor, through a standardization exercise, so as to ensure that the asset is always updated.^178^

This resolution stresses the necessity to design robust, durable and high-quality products,^179^ to manufacture long-lasting, repairable, upgradeable, recyclable products with interchangeable components^180^ and to boost the European labour market^181^ by enlightening consumers about product durability.^182^ These measures are essential to meet the objectives of the CE. Given the complex and multifaceted concept of planned obsolescence, further clarity at the EU level is required. For instance, an independent mechanism to test and detect the planned obsolescence would provide legal certainty whether objective or subjective lines of thought would be followed. The current test of ‘deliberate’ reduction of the product’s lifespan (currently employed by French law) has been heavily criticised by consumer and environmental associations, as producers can successfully argue that there was no intentional design choice.^183^

Most recently, the European Commission has taken some steps forward in its new Circular Economy Action Plan^184^ issued in 2020, which forms part of the main blocks of the European Green Deal,^185^ Europe’s new agenda for sustainable growth. Among other things, the Circular Economy Action Plan will propose a sustainable product policy legislative initiative, which has at its core the aim to widen the Ecodesign Directive beyond energy-related products to the broadest possible range of products by restricting single use and countering premature obsolescence. Disappointingly, ‘premature obsolescence’ is mentioned, and without further explanation, only three times throughout the document. Nevertheless, the Commission aims to present a ‘Circular Electronics Initiative’ mobilising existing and new instruments by promoting longer product lifetimes and embracing, *inter alia*, the following actions: imposing regulatory measures for electronics and information and communications technology (ICT), such as mobile phones, tablets and laptops, under the Ecodesign Directive so that devices are designed for energy efficiency and durability,^186^ reparability, upgradability, maintenance, reuse and recycling; and placing further focus on electronics and ICT as a priority sector for implementing the ‘right to repair’, including a right to update obsolete software. The Commission acknowledges that electrical and electronic equipment continues to be one of the fastest growing waste streams in the EU, with less than 40% being recycled. Therefore, it considers strengthening consumer protection by setting minimum requirements for sustainability labels/logos and for information tools.^187^

To conclude, it seems logical that the EU is directing its measures from an environmental side. This approach overlaps with the utilitarian thought in its focus on sustainability. It can also help to compensate for the shortcomings of supply-side and demand-side approaches to prevent planned obsolescence practices. However, it is doubtful whether ‘pockets of protection’ would be sufficient to deal with planned obsolescence, even with the plan to widen the scope of the Ecodesign Directive. In terms of making consumers more informed to address information asymmetry (and therefore market failure), there is still uncertainty in the rationality of their decision-making choices. On the one hand, under rational choice theory, which is based on the assumption that both consumers and producers have adequate information to consider all the consequences, including environmental concerns, in their decision making, when their benefits surpass the environmental issues (and they generally do), this does not offer a solution for planned obsolescence because they can be convinced that planned obsolescence is useful for them. On the other hand, in behavioural economics doctrine, consumers are irrational when making decisions; therefore, informing them would not provide a remedy. Although those theories can enlighten the ideas behind supply-side and demand-side perspectives, it does not cover environmental concerns. While there are an increasing number of discussions in consumer law and competition law about considering environmental aspects,^188^ the EU competition law model predominantly deals with economic objectives. Furthermore, neither consumer protection and unfair competition nor competition law adequately addresses the intergenerational argument, as the emphasis is on current rather than future consumers.

## 9. Concluding Remarks and the Way Forward

Different approaches are currently being undertaken to address planned obsolescence across various legislative measures, such as unfair competition and consumer protection, ecodesign and e-waste, and potentially competition law. The article has identified that these different measures are not without limits but are confined by various conditions. In most cases, the provisions require businesses to be transparent in communicating the expected lifespan of their products, instead of forcing businesses to build more durable products which could be easily upgraded or repaired. Yet, providing consumers with more technical information may lead to a confusopoly preventing them from making fully informed decisions. Furthermore, there is also uncertainty in the rationality of consumers’ decision-making choices. Dealing with planned obsolescence from either the demand side or the supply side is not sufficient, as the current provisions of unfair competition and consumer protection, and competition law are not adequately equipped to address it. Lengthening the lifespan of products is not only about contributing to the development of an economic model based on a balance of the needs between consumers and businesses, but also environmental imperatives, with due consideration given to intergenerational justice.^189^ Building on the CE concept, the article has embraced an environmental approach with the support of the utilitarian notion of responsibility for the sustainability of future generations to fulfil their needs. It has therefore argued that planned obsolescence should be banned by an EU measure to safeguard a fair playing field and achieve consistency across the EU Member States to ensure that business strategies are in conformity with a CE. This should be done regardless of whether specific practices are used by businesses to deliberately reduce the product lifespan (technological obsolescence) or they attempt to induce the public to replace goods which still retain substantial physical usefulness (style obsolescence). Additionally, durability should be in the DNA of product design, with further changes to eliminate the ‘take, make, throw-away’ culture by placing emphasis on repairability and reuse. Therefore, a three-tier approach with options inspired by the waste hierarchy could be introduced, where the most preferred option would be prevention (due to durable products), followed by boosting reparability and upgradability, with a further suggestion to promote trade-in options^190^ in order to influence recycling.

## Footnotes

1 Commission, ‘Closing the Loop—An EU Action Plan’ (Communication) COM (2015) 614 final.

2 Mariateresa Maggiolino, ‘Planned Obsolescence: A Strategy in Search of Legal Rules’ (2019) 50 International Review of Intellectual Property and Competition Law 405.

3 For instance, in the scenarios where software no longer works once an operating system is updated. This is also known as incompatibility obsolescence.

4 Glenn Adamson and David Gordon, *Industrial Strength Design: How Brooks Stevens Shaped Your World* (MIT Press 2003) 4–5.

5 Thomas Princen, *Treading Softly: Paths to Ecological Order* (MIT Press 2010).

6 According to the United Nations estimates elaborated by Worldometers, the world population in August 2020 reached 7.8 billion.

7 Matteo Zallio and Damon Berry, ‘Design and Planned Obsolescence. Theories and Approaches for Designing Enabling Technologies’ (2017) 20 Design Journal 3749; Pameet Singh and Peter Sandborn, ‘Obsolescence Driven Design Refresh Planning for Sustainment-Dominated Systems’ (2006) 51 Engineering Economist 115.

8 Roland Strausz, ‘Planned Obsolescence as an Incentive Device for Unobservable Quality’ (2009) 119(540) Economic Journal 1405; Michael Waldman, ‘Planned Obsolescence and the R&D Decision’ (2006) 27 Rand Journal of Economics 583; Barak Orbach, ‘The Durapolist Puzzle: Monopoly Power in Durable-Goods Market’ (2004) 21 Yale Journal on Regulation 67; Jeremy Bulow, ‘An Economic Theory of Planned Obsolescence’ (1986) 101 Quarterly Journal of Planned Obsolescence 729.

9 Julio Rivera and Amrine Lallmahomed, ‘Environmental Implications of Planned Obsolescence and Product Lifetime: A Literature Review’ (2016) 9 International Journal of Sustainable Engineering 119; Joseph Guiltinan, ‘Creative Destruction and Destructive Creations: Environmental Ethics and Planned Obsolescence’ (2009) 89 Journal of Business Ethics 19; Tim Cooper, *Longer Lasting Products: Alternatives to the Throwaway Society* (Gower 2010); Harald Wieser, ‘Beyond Planned Obsolescence: Product Lifespans and the Challenges to a Circular Economy’ (2016) 25(3) Ecological Perspectives on Science and Society 156.

10 Stefan Wrbka, ‘The Potential and Limits of Teleological Reduction Shown with the Example of the Austrian Warranty Regime’ in Mark Fenwick and Stefan Wrbka (eds), *Flexibility in Modern Business Law: A Comparative Assessment* (Springer 2016) 28; Maggiolino (n 2); Larry DiMatteo and Stefan Wrbka, ‘Planned Obsolescence and Consumer Protection: The Unregulated Extended Warranty and Service Contract Industry’ (2019) 28 Cornell Journal of Law and Public Policy 483.

11 Rosalie Koolhoven and Luc Heerema, ‘Fighting Planned Obsolescence or “The Lightbulb Conspiracy” as an Unfair Commercial Practice: For a Circular Economy’ in Maria Miguel Carvalho (ed), *Law and Technology: E Tec Yearbook* (JustGov and University of Minho 2018) 27–45; Eleonore Maitre-Ekern and Carl Dalhammar, ‘Regulating Planned Obsolescence: A Review of Legal Approaches to Increase Product Durability and Reparability in Europe’ (2016) 25 RECIEL 378.

12 Note: given that environmental law has a broad scope, this article will address some aspects, such as e-waste management, ecodesign and related aspects.

13 There have been no comprehensive studies placing planned obsolescence as some sort of ‘anticompetitive’ practice, except some limited editorial article presented by Maggiolino (n 2).

14 The issue of planned obsolescence is a natural consequence (and a negative externality) of capitalism, which triggers the number of commercial transactions, leading to increased consumption and, inevitably, waste. It affects every segment of society since the scope of planned obsolescence has already been extended by several industries, such as textile, electronics, automobiles, agriculture, printing and software. See Bulow, ‘An Economic Theory’ (n 8); Jesús Pineda, *Planned Obsolescence and the Rule of Law* (Universidad Externado de Colombia 2018).

15 Apart from electronics, the scope of planned obsolescence has already been extended by several industries (see n 14). See Bulow, ‘An Economic Theory’ (n 8); Pineda (n 14); Lisa J Hackett, ‘Addressing Rage: The Fast Fashion Revolt’ (2019) 22(1) Journal of Media and Culture; Tim Cooper, ‘Inadequate Life? Evidence of Consumer Attitudes to Product Obsolescence’ (2004) 27 Journal of Consumer Policy 421.

16 Giles Slade, *Made to Break: Technology and Obsolescence in America* (Harvard UP 2007) 3.

17 ibid 4–5.

18 Adolphe Chaillet invented a metallic filament for the light bulbs capable of lasting 1500 hours in 1895, which was increased to 2500 hours following further advancements.

19 Rivera and Lallmahomed (n 9) 119; Taiwo Aladeojebi, ‘Planned Obsolescence’ (2013) 4 International Journal of Scientific and Engineering Research 1504.

20 EI du Pont de Nemours & Company.

21 See Rivera and Lallmahomed (n 9) 119.

22 Bernard London, ‘Ending the Depression through Planned Obsolescence’ (1932) <https://babel.hathitrust.org/cgi/pt?id=wu.89097035273&view=1up&seq=7> accessed 6 November 2019

23 Slade (n 16) 72–3.

24 Theodor Adorno and Max Horkheimer, ‘The Culture Industry: Enlightenment as Mass Deception’ in Gunzelin Noerr (ed), *Dilaectic of Enlightenment: Philosophical Fragments* (Stanford UP 2002) 94–136.

25 Philip Kotler and others, *Principles of Marketing* (Pearson 2008) 77–9.

26 This slogan defining planned obsolescence was first used by Brooks Stevens. See Adamson and Gordon (n 4) 4.

27 Slade (n 16) 13–23.

28 Princen (n 5).

29 ibid.

30 Stephanie Boulos and others, ‘The Durability of Products: Final Report’ (European Commission Directorate-General Environment, Ricardo-AEA/R/ED59246, 17 August 2015) 7 <http://publications.europa.eu/resource/cellar/6c325b55-7352-11e5-86db-01aa75ed71a1.0001.01/DOC_1> accessed 28 August 2020.

31 The author refers to ‘purposeful obsolescence’. See Paul Gregory, ‘A Theory of Purposeful Obsolescence’ (1947) 14 Southern Economic Journal 24. See also Aladeojebi (n 19) 1504.

32 ibid.

33 Slade (n 16) 3–4.

34 Also known as contrived or built-in obsolescence. See Orbach (n 8) 91–2.

35 Gregory (n 31) 24.

36 Slade (n 16) 264.

37 In July 2017, the European Parliament urged the European Commission to take measures on planned obsolescence, which, *inter alia*, should include an EU-level definition. Further discussion is provided in section 8.

38 Adam Smith, *An Inquiry into the Nature and Causes of the Wealth of Nations*, Vol I (3rd edn, Methuen 1922) 58–9.

39 Jeffrey Herbener, ‘Ludwig von Mises and the Austrian School of Economics’ (1991) 5 Review of Austrian Economics 33.

40 Each product has a cluster of different attributes, such as price, quality and availability of related services.

41 Robert Lande and Neil Averitt, ‘Consumer Sovereignty: A Unified Theory of Antitrust and Consumer Protection Law’ (1997) 65 ALJ 716.

42 Dan Ariely, *Predictably Irrational: The Hidden Forces that Shape Our Decisions* (Harper Collins Publishers 2009); Daniel Kahneman, *Thinking, Fast and Slow* (Penguin Books 2011).

43 This notion acknowledges that humans use heuristics or short cuts in making quasi-rational decisions. See Amos Tversky and Daniel Kahneman, ‘Judgment under Uncertainty: Heuristics and Biases’ (1974) 185 Science 1124; Cass Sunstein, ‘Behavioral Analysis of Law’ (1997) 64 U Chi L Rev 1179.

44 Richard Thaler and Cass Sunstein, *Nudge: Improving Decisions About Health, Wealth, and Happiness* (Penguin Books 2008).

45 But it could be applied in a broader context.

46 Paolo Siciliani, Christine Riefa and Harriet Gamper, *Consumer Theories of Harm: An Economic Approach to Consumer Law Enforcement and Policy Making* (Hart Publishing 2019) 141.

47 ibid 142.

48 European Economic and Social Committee, ‘Towards More Sustainable Consumption: Industrial Product Lifetimes and Restoring Trust through Consumer Information’ (Opinion of the European Economic and Social Committee and the Committee, CCMI/112, Brussels, 17 October 2013).

49 Gregory (n 31) 24–45; Harald Wieser and Nina Tröger, ‘The Use-Time and Obsolescence of Durable Goods in the Age of Acceleration: An Empirical Investigation among Austrian Households’ (AK 2015) 3 <www.beuc.eu/documents/files/FC/durablegoods/articles/0515_AK_Austria.pdf> accessed 28 August 2020.

50 Maitre-Ekern and Dalhammar (n 11) 394–420.

51 See European Economic and Social Committee (n 48).

52 Tim Cooper, ‘The Value of Longevity: Product Quality and Sustainable Consumption’ (Global Research Forum on Sustainable Production and Consumption, Rio de Janeiro, 13–15 June 2012).

53 Raymond Vernon, ‘International Investment and International Trade in the Product Cycle’ (1996) 80 Quarterly Journal of Economics 190.

54 ibid 195–6.

55 Luis Cabral, *Introduction to Industrial Organization* (2nd edn, The MIT Press, 2017) 6.

56 Guriqbal Jaiya, ‘Role of the Intellectual Property System in Development and Marketing of New Products’ (WIPO) <www.wipo.int/export/sites/www/sme/en/documents/pdf/lifecycle.pdf> accessed 24 October 2019.

57 Bulow, ‘An Economic Theory’ (n 8) 729–49.

58 cf Slade (n 16).

59 Rudi Kurz, ‘Quality, Obsolescence and Unsustainable Innovation’ (2015) 28 Ekonomski Vjesnik/Econviews 511.

60 Orbach (n 8) 95–9.

61 Maximilian Kling, Ferdinand Zotz and Dana Huranova, ‘WEEE Compliance Promotion Exercise: Final Report’ (European Commission for Assistance to the Commission on Technical, Socio-economic and Cost-Benefit Assessments Related to the Implementation and Further Development of EU Waste Legislation, 21 December 2017) <https://publications.europa.eu/en/publication-detail/-/publication/09c7215a-49c5-11e8-be1d-01aa75ed71a1/language-en> accessed 28 August 2020.

62 *Denise Dantas and Lia Assumpção, ‘Planned Obsolescence, Consumer Practices and Design: A Survey on Consumer Goods’ (2017) 3* Mix Sustentável 191.

63 Eurostat, ‘Waste Statistics—Electrical and Electronic Equipment’ (May 2019) <https://ec.europa.eu/eurostat/statistics-explained/index.php/Waste_statistics_-_electrical_and_electronic_equipment> accessed 28 August 2020.

64 Commission, ‘Waste Electrical & Electronic Equipment (WEEE)’ <https://ec.europa.eu/environment/waste/weee/index_en.htm> accessed 28 August 2020.

65 European Union Network for the Implementation and Enforcement of Environmental Law, ‘New Circular Economy Report Focuses on Electrical and Electronic Equipment (EEE)’ (2019) <www.impel.eu/new-circular-economy-report-focuses-on-electrical-and-electronic-equipment-eee> accessed 28 August 2020.

66 Jae Park and others, ‘Effects of Electronic Waste on Developing Countries’ (2017) 2(128) Advances in Recycling and Waste Management 1.

67 Christine Cole and others, ‘An Assessment of Achievements of the WEEE Directive in Promoting Movement up to the Waste Hierarchy: Experiences in the UK’ (2019) 87 Waste Management 417.

68 Approximately 80% of the total e-waste is exported from developed countries to developing countries. Suthipong Sthiannopkao and others, ‘Handling e-Waste in Developed and Developing Countries: Initiatives, Practices, and Consequences’ (2013) 463–4 Science of the Total Environment 1147.

69 Jim Puckett, Chris Brandt and Hayley Palmer, ‘Holes in the Circular Economy: WEEE Leakage from Europe’ (Basel Action Network 2018) 3 <www.nvmp.nl/onderzoek/holes-in-the-circular-economy-weee-leakage-from-europe.html> accessed 28 August 2020.

70 Amit Kumar, Maria Holuszko and Denise Espinosa, ‘e-Waste: An Overview on Generation, Collection, Legislation and Recycling Practices’ (2017) 122 Resources, Conservation and Recycling 32; Berrin Tansel, ‘From Electronic Consumer Products to e-Wastes: Global Outlook, Waste, Quantities, Recycling Challenges’ (2017) 98 Environment International 35.

71 US Geological Survey, ‘National Minerals Information Center’ <www.usgs.gov/centers/nmic/lithium-statistics-and-information> accessed 28 August 2020.

72 Grace Livingstone, ‘The Farmers Who Worry about Our Phone Batteries’ (*BBC News*, 15 August 2019) <www.bbc.com/news/business-49355817> accessed 28 August 2020.

73 Commission, ‘Closing the Loop’ (n 1).

74 It should be noted that waste prevention is a preferred option in the waste hierarchy under art 4 of the Waste Framework Directive: Directive 2008/98/EC of the European Parliament and of the Council of 19 November 2008 on waste and repealing certain Directives [2008] OJ L312/3, as amended by Directive (EU) 2018/851 of the European Parliament and of the Council of 30 May 2018 amending Directive 2008/98/EC on waste.

75 Commission, ‘A New Circular Economy Action Plan: For a Cleaner and More Competitive Europe’ (Communication) COM (2020) 98 final. Further discussion is provided in section 8.

76 Jonathan Riley, ‘Utilitarianism and Economic Theory’ in Steven Durlauf and Lawrence Blume (eds), *The New Palgrave Dictionary of Economics* (Springer 2008) 6923–33. Utilitarianism also forms the intellectual basis of welfare economics. See John Smart and Bernard Williams, *Utilitarianism: For and Against* (CUP 1973) 140; Kenneth Arrow, ‘Some Ordinalist-Utilitarian Notes on Rawls’s Theory of Justice’ (1973) 70 Journal of Philosophy 246.

77 Jeremy Bentham, *Introduction to the Principles of Morals and Legislation* (Doubleday 1961) 273; John Mill, *Utilitarianism* (Dolphin 1961) 433–4. Mill added a quality element to Betham’s quantitative approach of the felicific calculus, suggesting that pain and pleasure are too subjective to be measured accurately.

78 Smart and Williams (n 76).

79 John Harsanyi, ‘Rule Utilitarianism and Decision Theory’ in Hans Gottinger and Werner Leinfellner (eds), *Decision Theory and Social Ethics: Issues in Social Choice* (D Reidel Publishing 1976) 3–33.

80 Kurz (n 59) 514. See also Ted Gayer and Emily Parker, ‘Cash for Clunkers: An Evaluation of the Car Allowance Rebate System’ (Economic Studies at Brookings 2013).

81 Bryan Norton, *The Preservation of Species* (Princeton UP 1986); Bryan Norton, *Toward Unity* (OUP 1991).

82 Axel Gosseries, ‘Theories of Intergenerational Justice: A Synopsis’ (2008) 1 Sapiens 61.

83 Joerg Tremmel, *A Theory of Intergenerational Justice* (Earthscan 2009) 58.

84 Edith Weiss, ‘Intergenerational Equity: A Legal Framework for Global Environmental Change’ in Edith Weiss (ed), *Environmental Change and International Law: New Challenges and Dimensions* (United Nations UP 1992) ch 12; United Nations Secretary-General, ‘Intergenerational Solidarity and the Needs of Future Generations’ (A/68/322, 15 August 2013); United Nations General Assembly, ‘Transforming Our World: The 2030 Agenda for Sustainable Development’ (Resolution 70/1, adopted 25 September 2015).

85 Peter Singer, ‘Famine, Affluence, and Morality’ (1972) 1(3) Philosophy & Public Affairs 231. Singer in this context modifies traditional utilitarianism, suggesting that minimising suffering is morally more important than maximising the satisfaction of ‘wants’.

86 Avner De-Shalit, *Why Posterity Matters: Environmental Policies and Future Generations* (Routledge 1995) 6–10. Similar is the concern about maximising future utility by thinking the intergenerational distribution of resources has also been brought under the sense of equity, which necessitates the needs of today *vis-à-vis* future generations. Wilfred Beckerman, ‘Debate: Intergenerational Equity and the Environment’ (1997) 5 Journal of Political Philosophy 397.

87 Additionally, repeat packaging of all those products also poses a danger for the environment. See Rivera and Lallmahomed (n 9) 122.

88 Directive 2005/29/EC of 11 May 2005 concerning unfair business-to-consumer commercial practices in the internal market (Unfair Commercial Practices Directive) [2005] OJ L149; Commission, ‘Guidance on the Implementation/Application of Directive 2005/29/EC on Unfair Commercial Practices’ SWD (2016) 163 final, s 3.4.8 on planned obsolescence.

89 Directive 2011/83/EU of 25 October 2011 on consumer rights [2011] OJ L304.

90 Directive (EU) 2019/771 of 20 May 2019 on certain aspects concerning contracts for the sale of goods [2019] OJ L136.

91 Jana Valant, ‘Planned Obsolescence: Exploring the Issue’ (European Parliament Think Tank 2016) 4 <www.europarl.europa.eu/thinktank/en/document.html?reference=EPRS_BRI%282016%29581999> accessed 28 August 2020.

92 Commission, ‘Attitudes of European Citizens towards Waste Management and Resource Efficiency’ (Report, Flash Eurobarometer 388, September 2014) 20 <https://ec.europa.eu/commfrontoffice/publicopinion/flash/fl_388_en.pdf> accessed 28 August 2020.

93 Directive 1999/44/EC on certain aspects of the sale of consumer goods and associated guarantees (Consumer Sales and Guarantees Directive) [1999] OJ L171, arts 5(1)–(3). This directive has been replaced by Directive (EU) 2019/771 (n 90).

94 ibid art 7(1).

95 24 out of 27 Member States incorporated related provisions in their national laws, whereas Bulgaria, Greece and Lithuania have not provided any provision going beyond the minimum harmonisation clauses of the CSGD. See Commission, ‘Article 8a of the Consumer Sales and Guarantees Directive’ <https://ec.europa.eu/info/article-8a-consumer-sales-and-guarantees-directive_en> accessed 28 August 2020.

96 Stefan Wrbka and Larry DiMatteo, ‘Comparative Warranty Law: Case of Planned Obsolescence’ (2019) 21 U Pa J Int’l Bus L 910.

97 Directive (EU) 2019/771 (n 90).

98 Joasia Luzak, ‘Status Quo on Fighting Planned Obsolescence’ (*Recent Developments in European Consumer Law*, 10 January 2019) <http://recent-ecl.blogspot.com/2019/01/status-quo-on-fighting-planned.html> accessed 28 August 2020.

99 Confirmed in Case C-611/14 *Canal Digital Danmark A/S* [2016] ECLI:EU:C 2016:800.

100 See Commission, ‘Guidance on the Implementation/Application of Directive 2005/29/EC’ (n 88) 8.

101 According to art 5, Annex I lists the commercial practices that should be regarded as unfair in all circumstances.

102 See eg Koolhoven and Heerema (n 11).

103 Unfair Commercial Practices Directive (n 88) art 7(1).

104 See eg *Canal Digital Danmark A/S* (n 99) para 24, which provided further clarification between invitations to purchase, misleading omissions and misleading actions in the context of the UCDP. Specifically, in this case, the court noted that when assessing whether a commercial practice must be considered as a misleading omission, consideration should be given to the context in which that practice takes place, namely the limitations of time and space imposed by the communications medium used, regardless of whether such a requirement is expressed in the national legislation.

105 Unfair Commercial Practices Directive (n 88) art 7.

106 See Koolhoven and Heereman (n 11) 38–9.

107 Valerio Romano, ‘The Law and Economics of Planned Obsolescence: A Transatlantic Antitrust Investigation’ (Stanford Law School 2018) <https://law.stanford.edu/projects/the-law-and-economics-of-planned-obsolescence-a-transatlantic-antitrust-investigation> accessed 28 August 2020.

108 Maggiolino (n 2).

109 ibid 405.

110 Steven Shugan, ‘Price–Quality Relationships’ (1984) 11 Advances in Consumer Research 627.

111 Romano (n 107).

112 Bulow, ‘An Economic Theory’ (n 8) 729; Orbach (n 8) 67; Ronald Coase, ‘Durability and Monopoly’ (1972) 15 JLE 143; Jeremy Bulow, ‘Durable-Good Monopolists’ (1982) 90 Journal of Political Economy 314; Jeremy Bulow and Lawrence Summers, ‘A Theory of Dual Labor Markets with Application to Industrial Policy, Discrimination, and Keynesian Unemployment’ (1986) 4 Journal of Labor Economics 376.

113 Orbach (n 8) 67; Chui Miao, ‘Tying, Compatibility and Planned Obsolescence’ (2010) 58 Journal of Industrial Economics 579, 604; Michael Waldman, ‘A New Perspective on Planned Obsolescence’ (1993) 108 Quarterly Journal of Economics 273; Waldman, ‘Planned Obsolescence’ (n 8) 583.

114 Drew Fudenberg and Jean Tirole, ‘Upgrades, Tradeins, and Buybacks’ (1998) 29 RAND Journal of Economics 235; Daniel Benjamin and Roger Kormendi, ‘The Interrelationship between Markets for New and Used Durable Goods’ (1974) 17 JLE 381.

115 Jay Choi, ‘Network Externality, Compatibility Choice, and Planned Obsolescence’ (1994) 42 Journal of Industrial Economics 167; Michael Waldman, ‘Durable Goods Pricing When Quality Matters’ (1996) 69 Journal of Business 489; Igal Hendel and Alessandro Lizzeri, ‘Adverse Selection in Durable Goods Markets’ (1999) 89 American Economic Review 1097; Waldman, ‘Planned Obsolescence’ (n 8) 583; Hodake Morita and Michael Waldman, ‘Durable Goods, Monopoly Maintenance, and Time Inconsistency’ (2004) 13 Journal of Economics & Management Strategy 273; Jae Nahm, ‘Durable-Goods Monopoly with Endogenous Innovation’ (2004) 13 Journal of Economics & Management Strategy 303; Paul Grout and In-Uck Park, ‘Competitive Planned Obsolescence’ (2005) 36 RAND Journal of Economics 596; Miao (n 113) 579, 604.

116 Cartels are strictly outlawed in the EU and its Member States as they cause increased prices.

117 First established in Case 322/81 *NV Nederlandsche Banden Industrie Michelin v Commission of the European Communities* [1983] ECR 3461, para 57.

118 Such abuse may, in particular, consist in: (i) directly or indirectly imposing unfair purchase or selling prices, or other unfair trading conditions; (ii) limiting production, markets or technical development to the prejudice of consumers; (iii) applying dissimilar conditions to equivalent transactions with other trading parties, thereby placing them at a competitive disadvantage; or (iv) making the conclusion of contracts subject to acceptance by the other parties of supplementary obligations which, by their nature or according to commercial usage, have no connection with the subject of such contracts.

119 Case C-457/10 P *AstraZeneca AB and AstraZeneca plc v European Commission* [2012] ECLI:EU:C:2012:770.

120 An aftermarket is traditionally described as a product or service that is complementary to and follows on from another, such as spare parts, consumables or maintenance services. Alison Jones and Brenda Sufrin, *EU Competition Law: Text, Cases, and Materials* (6th edn, OUP 2016) 302.

121 Inability to upgrade of products should be evaluated in the built-in obsolescence field.

122 Case No IV/34.330 *Pelikan International Corporation v Kyocera Corporation* [1995] SG(95) D/11872.

123 Case No IV/E 2/36.431 *Info-Lab Ireland v Ricoh Company Ltd* [1998].

124 *Eastman Kodak Co v Image Technical Services* [1992] 504 US Supreme Court 451.

125 Case AT.40220 *Qualcomm (Exclusivity payments)* [2018] OJ C269.

126 Case T-83/91 *Tetra Pak International SA v Commission of the European Communities* [1994] ECR II-00755.

127 Case COMP D3/38.044 *NDC Health/IMS: Interim Measures* [2002] 4 CMLR 111.

128 Case COMP/C-3/37.792 *Microsoft Corporation v Sun Microsystems, Inc* [2005] 4 CMLR 965. IBM was found to have abused its dominant position by forcing independent service providers through imposing unfair conditions regarding the supply of spare parts. IBM offered to be legally bound to duly exercising their commitments in 2011, which included ensuring the maintenance of the aftermarket competition for independent service providers.

129 Jones and Sufrin (n 120) 358.

130 *AstraZeneca* (n 119).

131 Commission Decision of 15 June 2005 relating to a proceeding under Article 82 of the EC Treaty and Article 54 of the EEA Agreement (Case COMP/A. 37.507/F3 *AstraZeneca*) [2005] OJ L332/744.

132 Richard Posner, *Antitrust Law* (2nd edn, University of Chicago Press 2001) 33–51; Massimo Motta, *Competition Policy Theory and Practice* (CUP 2004) 411; see also Douglas Melamed, ‘Exclusive Dealing Agreements and Other Exclusionary Conduct—Are There Unifying Principles?’ (2006) 73 ALJ 375.

133 Directive (EU) 2018/851 of 30 May 2018 amending the Directive 2008/98/EC on waste [2018] OJ L150.

134 Directive 2012/19/EU of 4 July 2012 on waste electrical and electronic equipment (WEEE) [2012] OJ L197, recital 6, art 1; see also Directive 2011/65/EU on the Restriction of the Use of Hazardous Substances (RoHS) [2011] OJ L174. The RoHS was adopted, which requires heavy metals such as lead, mercury, cadmium, and hexavalent chromium and flame retardants such as polybrominated biphenyls (PBB) or polybrominated diphenyl ethers (PBDE) to be substituted by safer alternatives to facilitate recycling and reduce emission.

135 Directive 2008/98/EC of 19 November 2008 on waste and repealing certain Directives [2008] OJ L312, art 14, preambles 1, 26.

136 ibid art 14.

137 Natalie Jones and Geert van Calster, ‘Waste Regulation’ in Emma Lees and Jorge Viñuales (eds), *The Oxford Handbook of Comparative Environmental Law* (OUP 2009) 620.

138 Commission, ‘Ecodesign Working Plan 2016–2019’ (Communication) COM (2016) 773 final.

139 The Directive covers all energy-related products sold in the domestic, commercial and industrial sectors, with the exception of all means of transport (covered by other legislation).

140 Yet, with the new developments, and new categories of A++ and A+++, it is less obvious to the consumer to make her choices, therefore the Commission is planning to gradually reintroduce the simpler A–G scale. Regulation (EU) 2017/1369 of 4 July 2017 setting a framework for energy labelling [2017] OJ L198.

141 Commission, ‘Regulation Laying Down Ecodesign Requirements 1 October 2019’ <https://ec.europa.eu/energy/en/regulation-laying-down-ecodesign-requirements-1-october-2019> accessed 28 August 2020.

142 Mauro Cordella, Felice Alfieri and Javier Vicente Sanfelix Forner, ‘Analysis and Development of a Scoring System for Repair and Upgrade of Products’ (Joint Research Centre of the European Commission’s Science and Knowledge Service 2019) <https://publications.jrc.ec.europa.eu/repository/bitstream/JRC114337/jrc114337_report_repair_scoring_system_final_report_v3.2_pubsy_clean.pdf> accessed 28 August 2020.

143 European Parliament Resolution of 4 July 2017 on a longer lifetime for products: benefits for consumers and companies [2016] 2016/2272(INI), OJ C334/60, para 30.

144 Maitre-Ekern and Dalhammar (n 11) 386.

145 Commission, ‘Independent Testing Programme on Premature Obsolescence’ (2017) <https://ec.europa.eu/info/funding-tenders/opportunities/portal/screen/opportunities/topic-details/ce-sc5-02-2018> accessed 28 August 2020.

146 For instance, s 377-3 of the German Commercial Code, Handelsgesetzbuch, regulates the liability of the seller to notify consumers regarding the hidden defects of products. It is usually assumed that the seller is aware of all hidden effects. Even though planned obsolescence could fall under this category of a hidden defect, the provision is still not specific enough to test how planned obsolescence could be evaluated.

147 European Economic and Social Committee, ‘Tackling Premature Obsolescence in Europe’ (2018) <www.eesc.europa.eu/en/agenda/our-events/events/tackling-premature-obsolescence-europe> accessed 28 August 2020.

148 Law no 2015-992 on Energy Transition for Green Growth. See also Anaïs Michel, ‘Product Lifetimes through the Various Legal Approaches within the EU Context: Recent Initiatives against Planned Obsolescence’ in Conny Bakker and Ruth Mugge (eds), *Product Lifetimes and the Environment* (Delft University of Technology and IOS Press 2017) 266–70.

149 Romain Mauger, ‘The Voluminous Energy Transition Legal Framework in France and the Question of Its Recognition as a Branch of Law’ (2018) 122 Energy Policy 499. See also Andreas Rüdinger, ‘The French Energy Transition Law for Green Growth: At the Limits of Governance by Objectives’ (2015) IDDRI Issue Brief. For further reading, see Ministry for the Ecological and Inclusive Transition of France, ‘Energy Transition for Green Grown Act: User Guide for the Act and Its Attendant Actions’ (2015) <www.ecologique-solidaire.gouv.fr/sites/default/files/Energy%20Transition%20for%20Green%20Growth%20Act%20in%20action%20-%20Regions%2C%20citizens%2C%20business%20%28%2032%20pages%20-%20juillet%202016%20-%20Versions%20anglaise%29.pdf> accessed 28 August 2020; Ségolène Royal, ‘Multiannual Energy Plan: Empowering Citizens, Local Authorities, Businesses and the Government to Work Together towards Common Goals’ (2016) <www.ecologique-solidaire.gouv.fr/sites/default/files/Synth%C3%A8se_EN_PPE.pdf> accessed 28 August 2020.

150 Code de la consummation (French Consumer Code), arts L441-2 and L213-14-1.

151 ibid art L441-2.

152 Isabelle de Foucaud, ‘Obsolescence programmée: une plainte déposée contre des fabricants d’imprimantes’ *Le Figaro* (21 September 2017) <www.lefigaro.fr/conso/2017/09/19/20010-20170919ARTFIG00093-obsolescence-programmee-une-plainte-deposee-contre-des-fabricants-d-imprimantes.php> accessed 28 August 2020. There were also class-action suits against HP for unfair business practices related to its inkjet printers’ use of ink cartridges. In a global settlement, HP promised to discontinue the use of unnecessary replacement messages and compensate the consumers in the class action for $5,000,000 for future purchases from HP. See Case No 5:05-cv-3580-JF *HP Inkjet Printer Litigation* [2011] WL 1158635. See also JD Supra, ‘In re: HP Inkjet Printer Litigation: Ninth Circuit Reverses and Remands Hewlett-Packard Coupon Settlement’ (3 June 2013) <jdsupra.com/legalnews/in-re-hp-inkjet-printer-litigation-nin-77516> accessed 28 August 2020.

153 The Direction générale de la concurrence, de la consommation et de la répression des fraudes (DGCCRF) is the authority responsible for the enforcement of competition legislation, consumer affairs and fraud control in France.

154 Le portail de l’Économie, des Finances, de l’Action et des Comptes publics, ‘Transaction avec le groupe APPLE pour pratique commerciale trompeuse’ (Press Release, 7 February 2020) <www.economie.gouv.fr/dgccrf/transaction-avec-le-groupe-apple-pour-pratique-commerciale-trompeuse> accessed 28 August 2020.

155 Sustainability for All, ‘The Battle against Planned Obsolescence’ <www.activesustainability.com/sustainable-development/battle-against-planned-obsolescence> accessed 28 August 2020.

156 Stefan Schridde, Christian Kreiß and Janis Winzer, ‘Geplante Obsoleszenz’ (Expert report on behalf of the Alliance 90/The Greens 2013) <https://menschengerechtewirtschaft.de/wp-content/uploads/2016/02/Geplante-Obsoleszenz-Nov-15.pdf> accessed 28 August 2020; Jürgen Reuß and Cosima Dannoritzer, *Kaufen für die Müllhalde: Das prinzip der geplanten obsoleszenz* (Orange Press 2013); Christian Kreiß, *Geplanter Verschleiss: Wie die Industrie uns zu immer mehr und immer schnellerem Konsum antreibt—und wie wir uns dagegen wehren* (Europa Verlag 2014); Stefan Schridde, *Murks? Nein Danke!: Was wir tun können, damit die Dinge besser werden* (Oekom 2014).

157 Jeremy White, ‘Apple Sued over Slowing Phones in France, Where Planned Obsolescence Is a Crime’ *The Independent* (28 December 2017) <www.independent.co.uk/life-style/gadgets-and-tech/apple-lawsuit-phones-slow-france-planned-obsolescence-legal-challenge-crime-a8132371.html> accessed 28 August 2020.

158 Autorità Garante della Concorrenza e del Mercato (AGCM) Case No PS11039 *Apple* [2018]; AGCM, Case No PS11009 *Samsung* [2018].

159 These practices both violated arts 20, 21, 22 and 24 of Legislative Decree n.206/2005 (Codice del Consumo), which regulate unfair business practices, specifically misleading practices.

160 AGCM, ‘PS11009-PS11039—Apple E Samsung Sanzionati, Aggiornamenti Software Hanno Procurato Gravi Inconvenienti E/O Ridotto Le Funzionalità Di Alcuni Cellulari’ (2018) <http://adusbeflivorno.it/apple-e-samsung-sanzionati-aggiornamenti-software-hanno-procurato-gravi-inconvenienti-e-o-ridotto-le-funzionalita-di-alcuni-cellulari> accessed 28 August 2020.

161 Commission, ‘Antitrust: Commission Opens Investigations into Apple’s App Store Rules’ (Press Release, 16 June 2020) <https://ec.europa.eu/commission/presscorner/detail/en/ip_20_1073> accessed 28 August 2020.

162 Decision 20-D-04 of 16 March 2020 regarding practices implemented in the Apple products distribution sector.

163 Namely, part 1, art 10, which deals with abuse of a dominant position. Even though Russia does not have any obligation to align its competition rules to the EU, its Law On the Protection of Competition was nonetheless signiﬁcantly inﬂuenced by EU competition law as well as German national competition rules. Evgeny Khokhlov, ‘The Current State of Russian Competition Law in the Context of its Harmonisation with EU Competition Law’ (2014) 5 Journal of European Competition Law & Practice 32.

164 Decisions of 20 February 2017 No АК/11838/17 and of 3 May 2017 No АК/30808/17.

165 Le portail de l’Économie, des Finances (n 154).

166 In conformity with arts 191, 192 and 193 TFEU, and with the reference to the goal of ensuring the prudent and rational utilisation of natural resources.

167 European Economic and Social Committee, ‘EESC Study on Planned Obsolescence’ (Factsheet, 29 March 2016) <www.eesc.europa.eu/sites/default/files/resources/docs/factsheet-en.pdf> accessed 28 August 2020.

168 European Economic and Social Committee, ‘Tackling Premature Obsolescence in Europe’ (n 147).

169 ibid.

170 Valant (n 91) 4.

171 Thierry Libaert, ‘The Functional Economy’ (Own-Initiative Opinion of the European Economic and Social Committee 2016) 3 <www.eesc.europa.eu/en/our-work/opinions-information-reports/opinions/functional-economy-own-initiative-opinion> accessed 28 August 2020.

172 Jean-Baptiste Ledoyen, ‘Conceptualising the Functional Service Economy’ (Master’s thesis, Aalto University 2015) 42–5.

173 Michal Len, ‘Briefing on Job Creation Potential in the Re-use Sector’ (2015) <www.rreuse.org/wp-content/uploads/Final-briefing-on-reuse-jobs-website-2.pdf> accessed 28 August 2020.

174 European Parliament, ‘Making Durable, Reparable Goods for Consumers and Tackling Planned Obsolescence (Press Release, 30 May 2017) <www.europarl.europa.eu/news/en/press-room/20170530IPR76313/making-durable-reparable-goods-for-consumers-and-tackling-planned-obsolescence> accessed 28 August 2020.

175 ibid.

176 European Parliament Resolution (n 143) para 32.

177 ibid para 30.

178 ibid paras 37–40.

179 ibid paras 1–8.

180 ibid paras 9–15.

181 ibid paras 16–26.

182 ibid paras 27–9.

183 For instance, the chip preventing functioning of printer cartridges at 20% capacity is designed to ensure better quality, as at that point there is a high risk of the ink being uneven on the page etc. For further reading, see Maitre-Ekern and Dalhammar (n 11) 387.

184 Commission, ‘A New Circular Economy Action Plan’ (n 75).

185 Commission, ‘A European Green Deal: Striving to Be the First Climate-Neutral Continent’ (2019) <https://ec.europa.eu/info/node/123797> accessed 28 August 2020.

186 European Commission, ‘A New Circular Economy Action Plan’ (n 75) point 3.2.

187 ibid.

188 Vanessa Mak and Evelyne Terryn, ‘Circular Economy and Consumer Protection: The Consumer as a Citizen and the Limits of Empowerment through Consumer Law’ (2020) 43 Journal of Consumer Policy 227; Suzanne Kingston, *Greening EU Competition Law and Policy* (CUP 2011); Simon Holmes ‘Climate Change, Sustainability, and Competition Law’ (2020) 8 Journal of Antitrust Enforcement 354.

189 Carlos Montalvo, David Peck and Elmer Rietveld, ‘A Longer Lifetime for Products: Benefits for Consumers and Companies’ (Committee on Internal Market and Consumer Protection for European Parliament 2016); Hans Bruyninckx, ‘Moving towards a Green, Circular Economy’ (2016) 25(3) GAIA 145; Wieser (n 9).

190 When there is an opportunity to return an ‘old’ product to gain credits towards purchasing a new product.

